# Supplementary figures and images for: Nano-scale architecture of blood-brain barrier tight-junctions
Source: eLife. 2021 Dec 24;10:e63253. doi: 10.7554/eLife.63253 (PMC8747500; doi:10.7554/eLife.63253)

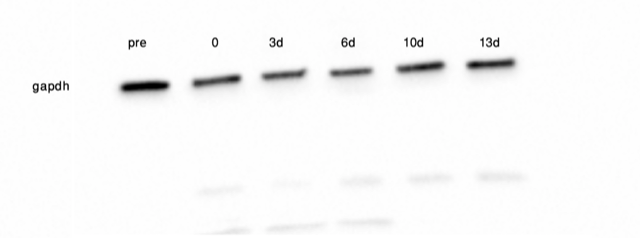

Supplement: Figure 2—figure supplement 1—source data 1. [file elife-63253-fig2-figsupp1-data1.zip › Figure 2-figure supplement 1- source data-gapdh.png]

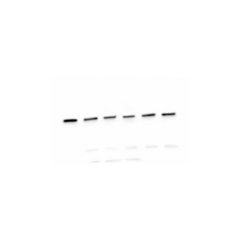

Supplement: Figure 2—figure supplement 1—source data 1. [file elife-63253-fig2-figsupp1-data1.zip › Figure 2-figure supplement 1- source data-gapdh-unlabled.tiff]

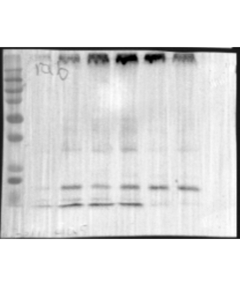

Supplement: Figure 2—figure supplement 1—source data 1. [file elife-63253-fig2-figsupp1-data1.zip › Figure 2-figure supplement 1-source data- cladn5-unlabled.tiff]

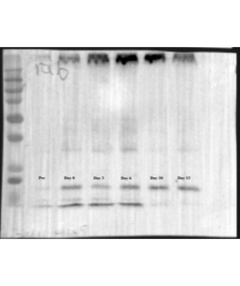

Supplement: Figure 2—figure supplement 1—source data 1. [file elife-63253-fig2-figsupp1-data1.zip › Figure 2-figure supplement 1-source data- cladn5 (1).tiff]
