# Supplementary material for: Nano-scale architecture of blood-brain barrier tight-junctions
Source: eLife. 2021 Dec 24;10:e63253. doi: 10.7554/eLife.63253 (PMC8747500; doi:10.7554/eLife.63253)
Supplement: Transparent reporting form [file elife-63253-transrepform1.pdf]

eLIFE
1st Floor
24 Hills Road
Cambridge CB2 1JP, UK
P 01223 855340
W elifesciences.org
T @elife

eLife's transparent reporting form

We encourage authors to provide detailed information *within their submission* to facilitate the interpretation and replication of experiments. Authors can upload supporting documentation to indicate the use of appropriate reporting guidelines for health-related research (see EQUATOR Network), life science research (see the BioSharing Information Resource), or the ARRIVE guidelines for reporting work involving animal research. Where applicable, authors should refer to any relevant reporting standards documents in this form.

If you have any questions, please consult our Journal Policies and/or contact us: editorial@elifesciences.org.

Sample-size estimation

- You should state whether an appropriate sample size was computed when the study was being designed
- You should state the statistical method of sample size computation and any required assumptions
- If no explicit power analysis was used, you should describe how you decided what sample (replicate) size (number) to use

Please outline where this information can be found within the submission (e.g., sections or figure legends), or explain why this information doesn't apply to your submission:

No explicit power analysis was used; sample size for all immunofluorescence experiments was determined empirically using standards generally employed by the field: a minimum of three animals per group in each experiment, a minimum of four tissue sections of each tissue and a minimum of 10 capillaries per group. Information of exact repetitions and sample numbers can be found in figure legends.

Replicates

- You should report how often each experiment was performed
- You should include a definition of biological versus technical replication
- The data obtained should be provided and sufficient information should be provided to indicate the number of independent biological and/or technical replicates
- If you encountered any outliers, you should describe how these were handled
- Criteria for exclusion/inclusion of data should be clearly stated
- High-throughput sequence data should be uploaded before submission, with a private link for reviewers provided (these are available from both GEO and ArrayExpress)

Please outline where this information can be found within the submission (e.g., sections or figure legends), or explain why this information doesn't apply to your submission:

eLife Sciences Publications, Ltd is a limited liability non-profit non-stock corporation incorporated in the State of Delaware, USA, with company number 5030732, and is registered in the UK with company number FC030576 and branch number BR015634 at the address 1st Floor, 24 Hills Road, Cambridge CB2 1JP | August 2014

Information of exact repetitions and sample numbers can be found in figure legends. In general:

For *in vitro* studies: 5 independent experiments were preformed in which every experiment was a different sub-culture passage preformed in a separate day (considered as a biological replicate). For technical replications (Figure 1), a minimum of 4 microscopic fields produced *n*=183 clusters (post-confluence) and 281 clusters (super-confluence). We excluded no outliers.
For in vivo studies: data from minimum three animals per group in each experiment were considered biological replicates. For technical replications a set of minimum 3 tissue sections of each animal produced:
1. Clustering properties (Figure 2, S1) - a set of 20 capillaries of each age (E12, P9), 657 clusters (E12) and 246 clusters (P9).
2. Total claudin-5 levels (Figure 2) - 25 capillaries (E12) and 27 capillaries (P9).
3. Molecular organization (Figure 3) - 40 capillaries.
4. Claudin-5 clusters that were coupled with ZO1 (Figure 3) - 40 clusters from 11 capillaries and 43 clusters from 11 capillaries (of 3 embryos/pups, P9 and E12 respectively).
5. Claudin-5 null mice set (Figure 4) - 109 capillaries of 4 wild-type embryos and 86 capillaries of 4 claudin-5 null embryos.
6. Tracer challenges (Figure 5) - 40 capillaries for each tracer.
We excluded no outliers.

Statistical reporting

- Statistical analysis methods should be described and justified
- Raw data should be presented in figures whenever informative to do so (typically when N per group is less than 10)
- For each experiment, you should identify the statistical tests used, exact values of N, definitions of center, methods of multiple test correction, and dispersion and precision measures (e.g., mean, median, SD, SEM, confidence intervals; and, for the major substantive results, a measure of effect size (e.g., Pearson's r, Cohen's d)
- Report exact p-values wherever possible alongside the summary statistics and 95% confidence intervals. These should be reported for all key questions and not only when the p-value is less than 0.05.

Please outline where this information can be found within the submission (e.g., sections or figure legends), or explain why this information doesn't apply to your submission:

Information of exact repetitions and sample numbers (raw data) is presented in figure legends, including statistical analysis methods of each experiment, definition of center (mean±s.e.m). These appear also in the Statistical analysis section in the methods section - All comparisons were performed by two-tailed Mann–Whitney U-tests, $P<0.05$ was considered significant (GraphPad Prism 8.0.1(244) for Windows, GraphPad Software, San diego, California, USA). Reports of exact p-values wherever possible appear in the text.

(For large datasets, or papers with a very large number of statistical tests, you may upload a single table file with tests, Ns, etc., with reference to sections in the manuscript.)

Group allocation

- Indicate how samples were allocated into experimental groups (in the case of clinical studies, please specify allocation to treatment method); if randomization was used, please also state if restricted randomization was applied
- Indicate if masking was used during group allocation, data collection and/or data analysis

Please outline where this information can be found within the submission (e.g., sections or figure legends), or explain why this information doesn't apply to your submission:

In tissues form wild-type mice samples were allocated based on the embryonic age (indicated in the text/methods and figure legends). In images from *in vitro* experiments samples were allocated based on the culture age (indicated in the text/methods and figure legends). In the data set of claudin-5 null and control littermates, the person collecting the data and analyzing was blind to the animal's genotype (in the Statistical analysis section in the methods section).

Additional data files ("source data")

- We encourage you to upload relevant additional data files, such as numerical data that are represented as a graph in a figure, or as a summary table
- Where provided, these should be in the most useful format, and they can be uploaded as "Source data" files linked to a main figure or table
- Include model definition files including the full list of parameters used
- Include code used for data analysis (e.g., R, MatLab)
- Avoid stating that data files are "available upon request"

eLIFE
1st Floor
24 Hills Road
Cambridge CB2 1JP, UK
P 01223 855340
W elifesciences.org
T @elife

Please indicate the figures or tables for which source data files have been provided:

NA

eLife Sciences Publications, Ltd is a limited liability non-profit non-stock corporation incorporated in the State of Delaware, USA, with company number 5030732, and is registered in the UK with company number FC030576 and branch number BR015634 at the address 1st Floor, 24 Hills Road, Cambridge CB2 1JP | August 2014
